# Possible Source Populations of the White-backed Planthopper in the Greater Mekong Subregion Revealed by Mitochondrial DNA Analysis

**DOI:** 10.1038/srep39167

**Published:** 2016-12-19

**Authors:** Xiang-yong Li, Dong Chu, Yan-qiong Yin, Xue-qing Zhao, Ai-dong Chen, Sathya Khay, Bounneuang Douangboupha, Mu Mu Kyaw, Manita Kongchuensin, Vien Vinh Ngo, Chung Huy Nguyen

**Affiliations:** 1 Agriculture Environment and Resources Institute, Yunnan Academy of Agricultural Sciences, Kunming 650205, China; 2 Key Lab of Integrated Crop Pest Management of Shandong Province, College of Agronomy and Plant Protection, Qingdao Agricultural University, Qingdao 266109, China; 3 Plant Protection Office, Cambodian Agricultural Research and Development Institute, Phnom Penh 01, Cambodia; 4 Horticulture Research Center, National Agriculture and Forestry Research Institute, Vientiane 7170, Lao PDR; 5 Department of Agricultural Research, Ministry of Agriculture and Irrigation, Nay Pyi Taw, Myanmar; 6 Plant Protection Research and Development Office, Department of Agriculture, Bangkok 10170, Thailand; 7 Plant Protection Research Institute, Vietnam Academy of Agricultural Sciences, Hanoi, Vietnam

## Abstract

The white-backed planthopper, *Sogatella furcifera* (Horváth) (Hemiptera: Delphacidae), is a serious pest of rice in Asia. However, little is known regarding the migration of this pest insect from the Greater Mekong Subregion (GMS) including Cambodia, Laos, Myanmar (Burma), Thailand, and Vietnam, into China’s Yunnan Province. To determine the migration patterns of *S. furcifera* in the GMS and putative secondary immigration inside China’s Yunnan Province, we investigated the population genetic diversity, genetic structure, and gene flow of 42 *S. furcifera* populations across the six countries in the GMS by intensive sampling using mitochondrial genes. Our study revealed the potential emigration of *S. furcifera* from the GMS consists primarily of three major sources: 1) the *S. furcifera* from Laos and Vietnam migrate into south and southeast Yunnan, where they proceed to further migrate into northeast and central Yunnan; 2) the *S. furcifera* from Myanmar migrate into west Yunnan, and/or central Yunnan, and/or northeast Yunnan; 3) the *S. furcifera* from Cambodia migrate into southwest Yunnan, where the populations can migrate further into central Yunnan. The new data will not only be helpful in predicting population dynamics of the planthopper, but will also aid in regional control programs for this economically important pest insect.

The white-backed planthopper, *Sogatella furcifera* (Horváth) (Hemiptera: Delphacidae), is a serious pest of rice in Asia^1^,^2^. *S. furcifera* can damage rice directly by feeding on the rice or indirectly by transmitting plant viruses such as southern rice black-streaked dwarf virus (SRBSDV)^3^,^4^,^5^. The planthopper can also be responsible for causing sudden and unexpected devastation to local rice crops due to their ability to annually migrate long distances^6^. Therefore, knowledge of the migration pattern and routes taken by the pest is important both for the control of the virus vector and the vectored virus^7^. Many previous studies, based on trajectory analyses and migration simulations, indicated that the East Asian populations of *S. furcifera* overwinter in Vietnam and southern Hainan Province, and in spring migrate to eastern China, Japan, and Korea, then migrate back to their overwintering areas in autumn^8^,^9^. Very little is known about the migration of this pest insect, however, in the Greater Mekong Subregion (GMS) which includes Cambodia, Laos, Myanmar (Burma), Thailand, Vietnam, and China’s Yunnan Province^7^.

*Sogatella furcifera* is one the most destructive pests of rice production in the GMS. The migration information of *S. furcifera* in the region, especially the immigration source into China’s Yunnan Province, has mainly been derived from trajectory analyses and migration simulations. Previous studies based on weather during the rice growth period and using the HYSPLIT method showed that although a small number of *S. furcifera* do successfully overwinter in China’s Yunnan Province, the immigrants from the spring migration are the major source of this pest. The source of the April to early May migration into Yunnan were estimated to have originated mainly in Myanmar, while immigrations occurring in mid-May were thought to have come from northern Vietnam^10^,^11^,^12^. Until now, there haven’t been any molecular markers available for this planthopper to help determine the migration source and the migration routes^7^.

It is difficult to determine potential migration source of the insect using conventional approaches such as atmospheric current analysis, fluorescent marker dyes, and radar monitoring primarily due to the small size of the insects coupled with their relatively short lifespan^13^,^14^. Approaches involving population genetics such as the use of mitochondrial and nuclear microsatellite markers can provide essential tools for overcoming these problems^15^,^16^,^17^,^18^,^19^,^20^. Previous study has demonstrated the genetic differentiation in *S. furcifera* from five disjunct localities in Korea, the Philippines, China (only two populations), Malaysia, and Vietnam using mitochondrial sequences. Genetic diversity of *S. furcifera* from Yunnan Province and three Southeastern Asia countries (Vietnam, Laos, and Myanmar) was also demonstrated using the inter-simple sequence repeat (ISSR) technique^6^. Although very useful from a genetics standpoint, these studies have not, however, substantially contributed to clarifying global migration patterns of the pest in the GMS.

In the present study, we investigated the genetic structure of 42 *S. furcifera* populations from across the six countries in the GMS by intensive sampling using mitochondrial genes. The objective of this study was to reveal the migration pattern of *S. furcifera* in the GMS and putative secondary immigration in China’s Yunnan Province. These results will provide insight into migrations of *S. furcifera* in the GMS countries, and should also form a basis for sustainable management of this economically important pest insect in this region.

## Results

### Mitochondrial COI haplotype network and distribution

**Figure 1:**
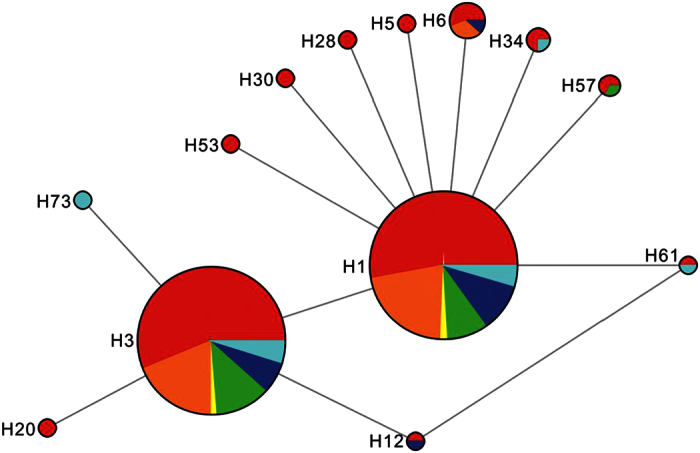
The haplotype network of the mitochondrial genes of COI. *The haplotype network of mtCOI genes were inferred using the median-joining algorithm 31 and the software, Network v.4.6.1.0 (Fluxus Technology Ltd, England). The Star contraction method and “Frequency.1” criterion were used for the calculations. After the calculation, the MP calculation was used to identify and remove unnecessary median vectors and links41. The network’s results were drawn and prepared using the software, Network Publisher v.2.0.0.1 (Fluxus Technology Ltd, England). Colors within the nodes: red, China’s Yunnan Province; orange, Laos; yellow, Thailand; green, Cambodia; blue, Vietnam; indigo, Myanmar.

The haplotype network tree of mtCOI formed two major groups (Fig. 1), which obviously displayed a star-like pattern with the most common haplotypes in the star’s center. Each of the dominant haplotypes was present in all of the GMS countries. A total of 73 mtCOI haplotypes (abbreviated as H1-H73, respectively) (Table S1) were identified in this study, among which 37 unique haplotypes were found in China’s Yunnan Province, and 23 in the other GSM countries. Thirteen haplotypes were shared by different populations.

Among the 13 shared haplotypes, seven haplotypes were shared by different populations from China’s Yunnan and other GSM countries as follows: 1) Haplotypes H1 and H3 were the dominant shared haplotypes in every population, occupying 75–100% in each population; 2) H6 was shared by the populations from Laos (L2 and L5 populations), Vietnam (V3 population), and China’s Yunnan (XP, YUJ, JP, and FN populations); 3) H12 was shared by the populations from Vietnam (V3 population) and China’s Yunnan (JP population); 4) H57 was shared by the populations from Cambodia (C2 population), and China’s Yunnan (GM and BS populations); 5) H34 was shared by the populations from Myanmar (M1 population), and China’s Yunnan (MS, MD, and SJ populations); 6) H61 was shared by the populations from Myanmar (M4 population) and China’s Yunnan (GM population).

Among the other six shared haplotypes, (except H73 which was only found in the M1 population from Myanmar), the remaining five haplotypes were shared only by populations within China’s Yunnan Province: 1) H30 was shared by Yunnan’s MS and SM populations; 2) H20 was shared by the province’s SJ and XP populations; 3) H5 was shared by the YUJ and YS populations; 4) H53 was shared by the JP and FN populations; and 5) H28 was shared by the ZY and KY populations.

### Mitochondrial COI diversity and genetic differentiation

Genetic diversity analysis based on mtCOI varied among different populations (Table 1). For example, the *Hd* of the populations from China’s Yunnan Province ranged from 0.297 to 0.725 while those from the other countries ranged from 0.173 to 0.830. The average *Hd* value from China’s Yunnan (0.5043) had no significant difference with those from the neighboring countries (0.4609) (*P* > 0.05). Thirty-one of the 38 populations had negative Fu’s *F* and Tajima’s *D* indices, suggesting a recent post-bottleneck population expansion^21^,^22^.

**Figure 2:**
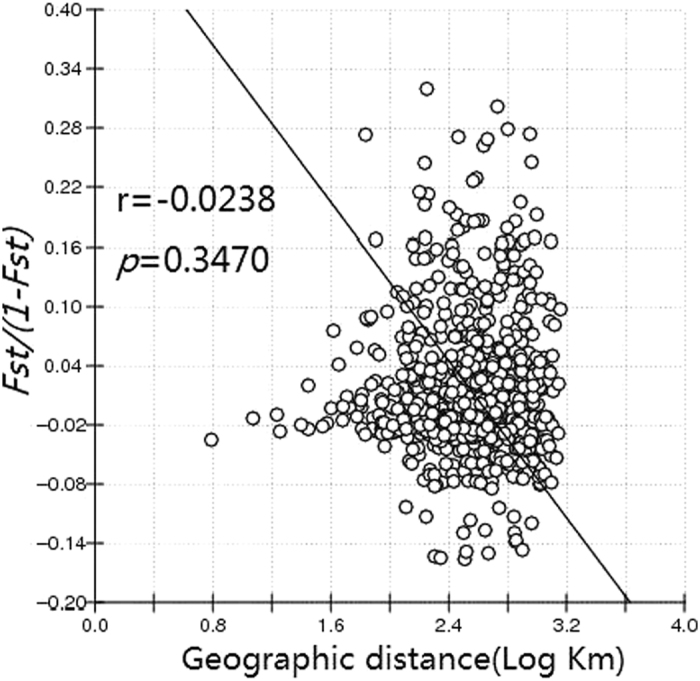
Relationship between genetic distance and log of geographical distance for pairwise population comparisons. *The line represents the regression line and circles represent the logarithm transformation of distance.

The pairwise *Fst* difference based on mitochondrial genes showed significant differentiation in 74 of the 861 population pairs (Table 2). Several populations, such as YS, FN, MD, MH, C2, C3, and M1, had more *Fst* values that were significantly different than other populations. The Mantel test results produced an r value of −0.0238 for mitochondrial genes (*P* = 0.3470) (Fig. 2), indicating that no correlation was found between genetic distance and geographical distance among the populations of *S. furcifera* in the GMS countries.

### Gene flow based on mitochondrial data

Analysis between each pair of the 42 populations showed the presence of high gene flow among different populations. Unidirectional estimates of *M* ranged from 28.6 (from M2 to T1) to 950.9 (from L4 to CX) (Table S2). When the *M* values were translated into effective migrants per generation (*N*_*e*_*m*) (Table 3), a high numbers of total migrants (*N*_*e*_*m* > 1000) in the GSM (excluding China’s Yunnan Province) were found in several populations, including L5 of Laos, C1-C3 of Cambodia, and V2 in Vietnam. The numbers of total migrants in Myanmar and other GSM localities, however, were relatively low.

In China’s Yunnan Province, a relatively high numbers of migrants (*N*_*e*_*m* > 1300) were found in a number of populations, including those from south Yunnan (e.g., JP), southwest Yunnan (e.g., MH), southeast Yunnan (e.g., YS), and central Yunnan (e.g., CX and YUJ), although, the numbers in some of the populations from these areas in Yunnan were very low, including KY in south Yunnan (*N*_*e*_*m* = 0.4), FN in southeast Yunnan (*N*_*e*_*m* = 0.7), GM in southeast Yunnan (*N*_*e*_*m* = 0.7), and SM in central Yunnan (*N*_*e*_*m* = 1.3). A number of the populations from western and northeastern Yunnan were also relatively low. This was especially true in the majority of those from western Yunnan where the numbers were quite low (*N*_*e*_*m* < 1.8) except in the MS population (*N*_*e*_*m* = 320.3). Interestingly, the average value for number of migrants into south Yunnan was the highest of all areas while that of west Yunnan was the lowest among all of the Yunnan populations.

## Discussion

### Evidence for intensive gene flow of *S. furcifera* in the GMS

The mtCOI haplotype network showed that the haplotypes H1 and H3 were widely distributed throughout the GMS, indicating the occurrence of extensive gene flow. The low ratio of population pairs having significant differentiation reinforces the postulation. In addition, the negative Fu’s *F* and Tajima’s *D* indices of most populations also demonstrated the occurrence of an extensive population expansion. These results are consistent with previous studies using trajectory analyses and migration simulations that the *S. furcifera* from the GMS countries can immigrate into China’s Yunnan Province^7^,^8^,^9^. Furthermore, the Mantel test results confirming that none of the populations of *S. furcifera* in the GMS are geographically isolated were also highly consistent with the postulated intensive gene flow.

However, the genetic diversity among different populations was fairly distinct and a number of migrants were also obviously different (Tables 1 and 3), suggesting that the amount of gene flow between different population varied. For example, the number of total migrants in the L5 population of Laos was higher than in the other populations from Laos.

### Potential migration sources of *S. furcifera* in the GMS

Based on shared mtCOI haplotypes, a close association was found to exist between: 1) the *S. furcifera* populations from southeast Yunnan, south Yunnan, Laos, and Vietnam; 2) the *S. furcifera* populations from Myanmar, west Yunnan, central Yunnan, and northeast Yunnan; 3) and the *S. furcifera* populations from Cambodia and southwest Yunnan. According to the seasonal weather data, we can infer that potential immigrations into Yunnan, China may occur in at least three main potential sources: 1) a source from Laos and Vietnam into adjacent southeastern and southern Yunnan; 2) a source from Myanmar into the adjacent western Yunnan, with subsequent migrations into central and northeastern Yunnan; 3) a source from Cambodia into southwestern Yunnan. For the potential source populations from Myanmar or Cambodia, they may be introduced into China’s Yunnan indirectly because of the long geographical distance, which should be further explored in the future researches.

In Yunnan Province, the haplotype composition of the populations in central Yunnan is closely linked with that from southeast Yunnan, west Yunnan, and southwest Yunnan since the H20, H30, and H5 haplotypes discovered in central Yunnan are also found in the latter three regions. When combined with the seasonal weather patterns, the populations in central Yunnan may have several sources such as from the neighboring regions. The haplotypes of the populations in south Yunnan are also closely associated with that found in northeast Yunnan, suggesting a potential immigration source from south Yunnan into northeast Yunnan.

Based on the combined data mentioned above, the potential emigration of *S. furcifera* from the GMS consists primarily of three major sources: 1) the *S. furcifera* from Laos and Vietnam migrate into south and southeast Yunnan, where they proceed to further migrate into northeast and central Yunnan; 2) the *S. furcifera* from Myanmar migrate into west Yunnan, and/or central Yunnan, and/or northeast Yunnan; 3) the *S. furcifera* from Cambodia migrate into southwest Yunnan, where the populations can migrate further into central Yunnan.

These postulated multiple sources of the *S. furcifera* populations in Yunnan, China are consistent with previous studies. Based on their analysis using the HYSPLIT model, Shen *et al*.^9^ concluded that the main source areas of the early migration of *S. furcifera* into Yunnan, China in 2009 were located in Myanmar, and that secondary source areas were in Laos and Vietnam, with a few coming from the Imphal area in Manipur, India. The frequent westerly winds coupled with the tremendous increase in rice production in the Myanmar source areas were considered to be the principal reasons causing the mass migration of *S. furcifera* into Yunnan Province^9^. Jiang *et al*.^10^ found similar results confirming that the main source areas of the early migration of *S. furcifera* in May were located in the middle of Myanmar, and secondarily in northern Thailand and Vietnam. The westerly and southwesterly wind carried massive numbers of *S. furcifera* into Funing (FN), a site in southeastern Yunnan. Wind shear combined with the occurrence of threshold flying temperatures associated with rainfall, caused a mass descent of *S. furcifera*. An analysis by Zheng *et al*.^12^ also pointed out that the source areas of an early April migration into Shizong (SZ), a site in northeastern Yunnan were mainly located in northeastern Myanmar and that secondary source areas were in northern Vietnam and the Golden Triangle area of GMS.

Our study not only reveals the potential migratory sources from Myanmar, Vietnam, and Laos, but also adds Cambodia as another source of *S. furcifera* migrants. This data will be helpful in predicting the population dynamics of this economically important planthopper, and will aid in regional control of this major pest insect.

### Future research on the *S. furcifera* in the GMS

The genetic diversity of species is closely associated with their ecological adaptations, which have been explored in many species especially in invasive alien species^19^,^23^,^24^. In this study, unique mtCOI haplotypes have been identified in many populations from the GMS, which indicates that the adaptation of these haplotypes are a response to the local environment including the unique climate, host plants, and agricultural activities experienced by the population from that particular region. The widespread mtCOI haplotypes may have robust adaptive abilities to the diverse ecological factors. On the other hand, the genetic diversity based on mitochondrial DNA may be inconsistent with that based on nuclear DNA. For example, a relatively high nuclear genetic diversity was revealed in introduced populations of the invasive species, *Bemisia tabaci* biotype Q in Shandong, China, while the mitochondrial genetic diversity was considerably lower^23^, suggesting that mitochondrial DNA may not be indicative of the level of diversity in the nuclear DNA. Therefore, more attention should be focused on the genetic diversity in nuclear DNA, which will help us further understand the relationship between genetic variation and ecological adaptation.

Although, based on mtCOI data, it is apparent that migrations from and within the GMS consist of many sources, detailed migration patterns may be more complex than expected due to variations of yearly ecological factors occurring in this region. In addition, recent studies have revealed the differences resulting from using mitochondrial and nuclear markers^18^,^23^,^25^,^26^. The combining molecular markers of distinct modes of inheritance, such as the combination of the mitochondrial and nuclear markers, can provide extra, complementary information on gene flow^27^,^28^. Further studies, including application of the nuclear markers, may lead to more effective research into migration pattern and population dynamics in various geographic regions, which will be essential in developing sustainable pest management strategies.

## Conclusions

The genetic diversity and structures found in the *S. furcifera* populations analyzed in this study enabled us to infer the planthopper’s migration sources. Based on our results, we can speculate that the potential migration of *S. furcifera* from the GMS consisted primarily of three major sources: 1) the *S. furcifera* from Laos and Vietnam migrate into southern and southeastern Yunnan. These populations can later migrate into northeast and central Yunnan; 2) the *S. furcifera* originating from Myanmar migrate into western Yunnan, and/or central Yunnan, and/or northeastern Yunnan; 3) the *S. furcifera* from Cambodia migrates into southeastern Yunnan, where the populations can further migrate into central Yunnan. Our study not only reaffirmed the detailed potential migration source from Myanmar, Vietnam, and Laos, but also demonstrated the migration of *S. furcifera* from Cambodia. The added data will be helpful in predicting the population dynamics of this pest insect, which, in turn will be useful in regional control programs for the planthopper.

## Materials and Methods

### Field sampling and DNA extraction

**Figure 3:**
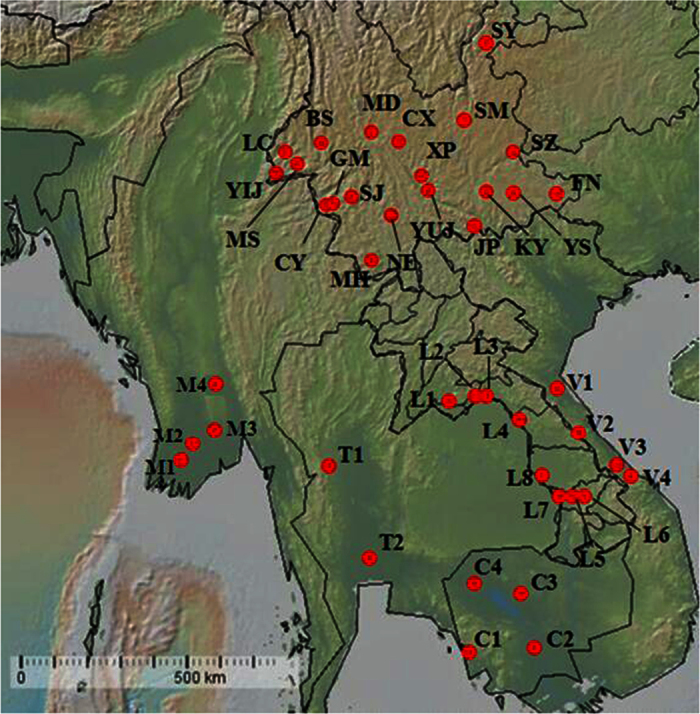
Geographical distribution of *Sogatella furcifera* populations in the Greater Mekong Subregion (GMS). *The map of Southeast Asia’s mainland was modified from the map generated using GeoMapApp (version 2) (http://www.geomapapp.org/).

Adult *S. furcifera* samples were collected from 42 locations in the GMS during 2014–2015 (Fig. 3; Table 4). The samples included 20 populations from China’s Yunnan Province, four from Vietnam, eight from Laos, two from Thailand, four from Cambodia, and four from Myanmar. The specimens were fixed in 95% ethanol and stored at −20 °C until DNA was extracted. Genomic DNA was individually extracted from each adult planthopper using the DNAzol kit (Molecular Research Center, Inc., Cincinnati, OH) and stored at −20 °C.

### Mitochondrial COI amplification and sequencing

The cytochrome coxidase subunit I gene of the mitochondrial DNA (mtCOI) was used to determine the genetically distinct populations of *S. furcifera.* All individual DNA samples were first amplified using the mtCOI primers CI-J-2183 (5′-CAACATTTATTTTGATTTTTTGG-3′) and L2-N-3014 (5′-TCCAATGCACTAATC TGCCATATTA-3′) and then sequenced^29^. The PCR reactions were performed in 20 μL buffer containing 2 μL 10× buffer, 1.5 mM MgCl_2_, 0.2 μM dNTPs, 1 unit Taq DNA polymerase, 2 μL template DNA, and 0.2 μM of each primer. PCR amplification was carried out as follows: initial denaturation at 94 °C for 5 min, followed by 35 cycles each of 30 s at 94 °C, 30 s at 50 °C, and 60 s at 72 °C, and a final elongation step at 72 °C for 30 min.

### Mitochondrial COI haplotype analysis

These sequences of mtCOI were aligned with Clustal W^30^ and were then checked for indels and numts. The haplotype network of mtCOI genes was inferred using the median-joining algorithm^31^. All calculations were conducted using the software program Network v.4.6.1.0 (Fluxus Technology Ltd., England).

The genetic diversity indices of each population which were analyzed based on mtCOI using DnaSP v.5.0^32^ included the number of polymorphic (segregating) sites (*S*), the total number of mutations (*η*)^33^, the average number of nucleotide differences (*K*)^34^, the number of haplotypes (H), the haplotype diversity (*Hd*)^35^, the nucleotide diversity (*π*)^35^, the nucleotide diversity with Jukes and Cantor correction (*π* (JC))^36^, and the number of net nucleotide substitutions per site between populations with Jukes and Cantor correction, *Da* (JC)^35^. Tajima’s *D (D*)^21^ and Fu ‘s *F* test^22^ were performed to detect deviation from neutrality.

The Weir and Cockerham’s fixation index *Fst*^37^, the traditional population differentiation approach, was calculated using ARLEQUIN v.3.5 software^38^. The correlation between genetic differentiation and geographic distance was examined using the Mantel test with IBDWS v.3.15 software^39^.

### Gene flow analysis based on mitochondrial COI data

The dispersal of different *S. furcifera* populations in the GMS, was determined by calculating the effective numbers of migrants per generation *N*_*e*_*m* using mitochondrial COI data. *N*_*e*_*m* is ϴ*M* (ϴ = *N*_*e*_μ, where μ is the mutation rate per site per generation; *M* = *m*/μ, where *m* is the migration rate) calculated using Bayesian search strategies in MIGRATE v. 3.2.16 software^40^.

## Additional Information

**How to cite this article**: Li, X.-y. *et al*. Possible Source Populations of the White-backed Planthopper in the Greater Mekong Subregion Revealed by Mitochondrial DNA Analysis. *Sci. Rep.* **6**, 39167; doi: 10.1038/srep39167 (2016).

**Publisher's note:** Springer Nature remains neutral with regard to jurisdictional claims in published maps and institutional affiliations.

## Supplementary Material

Supplementary Legends

## Figures and Tables

**Table 1:** Genetic diversity indices of *Sogatella furcifera* populations in the Greater Mekong Subregion (GMS).

Population code* (Number of individuals tested)	*S*	*η*	*H*	*D (p*)	*Fs (p*)	*Hd* (SD)	*π* (SD)	*K*	*π* (JC)
JP (25)	6	6	7	−1.38476 (0.07100)	**−3.67776 (0.00000)**	0.617 (0.098)	0.00145 (0.00031)	0.853	0.00145
KY (25)	7	7	7	**−1.59097 (0.03500)**	**−3.47179 (0.00200)**	0.617 (0.098)	0.00153 (0.00036)	0.900	0.00153
YS (24)	3	3	4	−0.37127 (0.35600)	−0.69024 (0.25300)	0.598 (0.057)	0.00116 (0.00019)	0.685	0.00116
FN (23)	3	3	4	−1.01220 (0.19300)	−1.48134 (0.04600)	0.447 (0.118)	0.00083 (0.00025)	0.490	0.00083
MD (25)	3	3	4	−1.50407 (0.05600)	**−2.44153 (0.00100)**	0.297 (0.115)	0.00053 (0.00022)	0.313	0.00053
BS (23)	5	5	5	−1.22178 (0.10300)	−1.54985 (0.08300)	0.609 (0.082)	0.00134 (0.00031)	0.791	0.00134
MS (19)	5	5	6	−1.43184 (0.05400)	**−3.17970 (0.00200)**	0.643 (0.108)	0.00131 (0.00030)	0.772	0.00131
YIJ (21)	5	5	5	**−1.79547 (0.01700)**	−2.59252 (0.00500)	0.424 (0.131)	0.00095 (0.00036)	0.562	0.00096
LC (23)	1	1	2	0.18585 (0.75800)	0.61246 (0.46500)	0.300 (0.105)	0.00051 (0.00018)	0.300	0.00051
NE (23)	1	1	2	0.53502 (0.83700)	0.87901 (0.52200)	0.356 (0.100)	0.00060 (0.00017)	0.356	0.00060
MH (25)	4	4	4	−0.76354 (0.25800)	−0.41635 (0.32600)	0.597 (0.054)	0.00129 (0.00027)	0.760	0.00129
SJ (24)	5	5	6	−1.24330 (0.11600)	**−2.81348 (0.01200)**	0.616 (0.091)	0.00130 (0.00027)	0.764	0.00130
GM (23)	5	5	6	−1.41008 (0.08700)	**−3.15734 (0.00300)**	0.601 (0.101)	0.00119 (0.00026)	0.704	0.00120
CY (25)	1	1	2	0.72124 (0.84900)	1.03183 (0.53000)	0.380 (0.091)	0.00065 (0.00016)	0.380	0.00065
CX (23)	4	4	5	−0.89102 (0.23000)	−1.74242 (0.08000)	0.581 (0.093)	0.00125 (0.00027)	0.735	0.00125
SM (24)	5	5	6	**−1.54913 (0.03900)**	**−3.50173 (0.00300)**	0.543 (0.111)	0.00106 (0.00027)	0.623	0.00106
XP (23)	3	3	5	−1.01220 (0.15600)	**−2.86701 (0.00200)**	0.391 (0.125)	0.00083 (0.00030)	0.490	0.00083
YUJ (23)	5	5	6	**−1.82093 (0.01600)**	**−4.26158 (0.00000)**	0.458 (0.126)	0.00087 (0.00028)	0.514	0.00087
ZY (23)	3	3	4	−0.73946 (0.25800)	−1.11359 (0.10300)	0.486 (0.105)	0.00098 (0.00026)	0.577	0.00098
SZ (24)	8	8	8	**−1.55882 (0.03700)**	**−4.07697 (0.00100)**	0.725 (0.073)	0.00187 (0.00036)	1.101	0.00187
L1 (25)	2	2	3	−0.12151 (0.43300)	−0.05780 (0.36500)	0.440 (0.095)	0.00085 (0.00021)	0.500	0.00085
L2 (24)	4	4	4	−0.71822 (0.25800)	−0.36644 (0.34400)	0.591 (0.081)	0.00134 (0.00033)	0.790	0.00134
L3 (21)	3	3	3	−1.45676 (0.07700)	−0.72758 (0.22200)	0.267 (0.120)	0.00063 (0.00033)	0.371	0.00063
L4 (19)	1	1	2	0.41712 (0.76900)	0.75823 (0.47800)	0.351 (0.111)	0.00060 (0.00019)	0.351	0.00060
L5 (17)	2	2	3	−1.06916 (0.19800)	−1.03838 (0.05600)	0.324 (0.136)	0.00057 (0.00026)	0.338	0.00057
L6 (22)	5	5	6	−1.28369 (0.09300)	**−2.89198 (0.00700)**	0.580 (0.111)	0.00132 (0.00033)	0.779	0.00133
L7 (22)	1	1	2	−0.64112 (0.24200)	−0.17575 (0.18500)	0.173 (0.101)	0.00029 (0.00017)	0.173	0.00029
L8 (19)	2	2	3	0.10134 (0.62600)	0.08500 (0.45200)	0.556 (0.073)	0.00101 (0.00018)	0.596	0.00101
C1 (24)	3	3	3	−0.76831 (0.21000)	0.12791 (0.44100)	0.409 (0.103)	0.00095 (0.00034)	0.558	0.00095
C2 (17)	2	2	3	0.04791 (0.61500)	0.01788 (0.39000)	0.559 (0.083)	0.00102 (0.00020)	0.603	0.00102
C3 (16)	4	4	4	−0.77113 (0.24700)	−0.47280 (0.34100)	0.642 (0.081)	0.00154 (0.00040)	0.908	0.00155
C4 (23)	2	2	3	−0.40840 (0.33600)	−0.34481 (0.27400)	0.423 (0.104)	0.00075 (0.00020)	0.443	0.00075
V1 (22)	2	3	4	−0.54947 (0.33300)	**−1.89698 (0.03100)**	0.398 (0.122)	0,.00071 (0.00023)	0.416	0.00071
V2 (16)	4	4	5	**−1.54972 (0.04200)**	**−2.75106 (0.00100)**	0.533 (0.142)	0.00103 (0.00033)	0.608	0.00103
V3 (23)	4	4	5	−1.07287 (0.18000)	−2.01641 (0.05100)	0.545 (0.104)	0.00113 (0.00028)	0.664	0.00113
V4 (21)	2	2	3	−0.84329 (0.22400)	−0.82277 (0.18700)	0.267 (0.120)	0.00060 (0.00029)	0.352	0.00060
M1(18)	8	8	8	−1.38547(0.07300)	**−3.87433(0.00200)**	0.830 (0.064)	0.00237 (0.00042)	1.399	0.00238
M2(12)	1	1	2	0.54055(0.80900)	0.73482(0.47900)	0.409 (0.133)	0.00069 (0.00023)	0.409	0.00070

^*^The indices of the T1, T2, M3, and M4 were not calculated because the number of individuals was below 10; *S*, number of polymorphic (segregating) sites; *η*, total number of mutations; *H*, number of haplotypes; *Hd*, haplotype diversity; *π*, nucleotide diversity; *K*, average number of nucleotide differences; *π*(JC), nucleotide diversity with Jukes and Cantor correction; *D*, Tajima’s *D* statistic; *Fs*, Fu ‘s *F* test statistic; *p*, Significance values of the parameters were evaluated using 10000 simulations.

**Table 2:** Pairwise *Fst* values for populations in the Greater Mekong Subregion (GMS).

	JP	KY	YS	FN	MD	BS	MS	YIJ	LC	NE	MH	SJ	GM	CY	CX	SM	XP	YUJ	ZY	SZ	L1	L2	L3	L4	L5	L6	L7	L8	T1	T2	C1	C2	C3	C4	V1	V2	V3	V4	M1	M2	M3	M4
JP	0.0000																																									
KY	−0.0203	0.0000																																								
YS	−0.0011	0.0154	0.0000																																							
FN	0.0210	0.0168	**0.1392**	0.0000																																						
MD	**0.0723**	0.0449	**0.2075**	−0.0036	0.0000																																					
BS	−0.0231	−0.0255	−0.0003	0.0294	**0.0664**	0.0000																																				
MS	−0.0084	−0.0164	0.0547	−0.0044	0.0068	-−0.0131	0.0000																																			
YIJ	0.0428	0.0262	**0.1564**	−0.0242	−0.0147	0.0412	0.0004	0.0000																																		
LC	0.0080	−0.0078	0.1129	−0.0150	−0.0026	0.0016	−0.0244	−0.0097	0.0000																																	
NE	−0.0118	−0.0210	0.0697	−0.0033	0.0239	−0.0174	−0.0283	0.0073	−0.0392	0.0000																																
MH	0.0036	0.0249	−0.0296	**0.1405**	**0.2145**	0.0139	0.0649	**0.1612**	**0.1233**	0.0813	0.0000																															
SJ	−0.0219	−0.0234	0.0109	0.0241	**0.0529**	−0.0267	−0.0213	0.0355	−0.0039	−0.0209	0.0207	0.0000																														
GM	−0.0130	−0.0158	0.0539	−0.0023	0.0172	−0.0185	−0.0231	0.0040	−0.0253	−0.0301	**0.0647**	−0.0156	0.0000																													
CY	−0.0169	−0.0230	0.0529	0.0078	0.0413	−0.0219	−0.0251	0.0207	−0.0298	−0.0421	0.0650	−0.0245	−0.0274	0.0000																												
CX	−0.0242	−0.0223	−0.0126	0.0551	0.1029	−0.0242	−0.0007	0.0696	0.0252	−0.0019	−0.0044	−0.0220	−0.0014	−0.0104	0.0000																											
SM	0.0110	−0.0024	**0.0945**	−0.0110	−0.0029	0.0049	−0.0252	−0.0092	−0.0283	−0.0238	**0.1054**	0.0009	−0.0171	−0.0164	0.0239	0.0000																										
XP	0.0307	0.0168	**0.1392**	−0.0208	−0.0036	0.0294	−0.0044	−0.0160	−0.0150	−0.0033	**0.1446**	0.0130	−0.0023	0.0078	0.0551	−0.0110	0.0000																									
YUJ	0.0516	0.0286	**0.1661**	−0.0213	−0.0156	0.0496	0.0057	−0.0227	−0.0064	0.0129	**0.1753**	0.0433	0.0092	0.0273	0.0798	−0.0060	−0.0135	0.0000																								
ZY	−0.0210	−0.0288	0.0297	0.0130	0.0463	−0.0306	−0.0224	0.0245	−0.0179	−0.0323	0.0407	−0.0287	−0.0234	−0.0340	−0.0179	−0.0100	0.0130	0.0317	0.0000																							
SZ	−0.0005	0.0061	−0.0248	**0.1058**	**0.1568**	−0.0007	0.0397	**0.1192**	**0.0813**	0.0501	−0.0225	0.0085	0.0377	0.0392	−0.0101	**0.0733**	**0.1058**	**0.1279**	0.0219	0.0000																						
L1	−0.0240	−0.0246	0.0212	0.0251	0.0656	−0.0272	−0.0183	0.0394	−0.0101	−0.0298	0.0326	−0.0278	−0.0202	−0.0338	−0.0229	−0.0027	0.0251	0.0475	−0.0320	0.0163	0.0000																					
L2	−0.0270	−0.0140	−0.0016	0.0289	**0.0922**	−0.0191	−0.0004	0.0523	0.0220	−0.0013	−0.0020	−0.0174	−0.0006	−0.0080	−0.0217	0.0217	0.0394	0.0624	−0.0144	−0.0013	−0.0180	0.0000																				
L3	0.0527	0.0285	**0.1768**	−0.0105	−0.0208	0.0466	0.0014	−0.0188	−0.0136	0.0080	**0.1843**	0.0398	0.0040	0.0234	0.0793	−0.0120	−0.0105	−0.0186	0.0282	**0.1307**	0.0453	**0.0704**	0.0000																			
L4	−0.0152	−0.0252	0.0684	−0.0111	0.0149	−0.0209	−0.0340	−0.0017	−0.0459	−0.0503	0.0793	−0.0245	−0.0353	−0.0460	−0.0045	−0.0304	−0.0111	0.0038	−0.0360	0.0459	−0.0326	−0.0045	−0.0008	0.0000																		
L5	0.0237	0.0089	**0.1500**	−0.0465	−0.0250	0.0247	−0.0152	−0.0386	−0.0311	−0.0128	0.1516	0.0184	−0.0132	0.0013	0.0550	−0.0256	−0.0336	−0.0369	0.0070	**0.1046**	0.0223	0.0324	−0.0303	−0.0211	0.0000																	
L6	−0.0252	−0.0220	0.0193	0.0093	0.0466	−0.0236	−0.0195	0.0254	−0.0108	−0.0249	0.0293	−0.0239	−0.0193	−0.0270	−0.0190	−0.0057	0.0154	0.0327	−0.0272	0.0122	−0.0277	−0.0153	0.0289	−0.0290	0.0086	0.0000																
L7	0.0655	0.0368	**0.2131**	−0.0118	−0.0302	0.0603	0.0074	−0.0220	−0.0158	0.0153	**0.2179**	0.0516	0.0085	0.0345	0.1000	−0.0127	−0.0118	−0.0232	0.0399	**0.1525**	0.0608	**0.0876**	−0.0306	0.0070	−0.0363	0.0397	0.0000															
L8	−0.0295	−0.0205	−0.0256	0.0740	**0.1370**	−0.0283	0.0040	0.0904	0.0436	0.0070	−0.0176	−0.0252	0.0025	−0.0056	−0.0361	0.0341	0.0740	**0.1027**	−0.0174	−0.0215	−0.0244	−0.0274	0.1082	0.0058	0.0824	−0.0204	**0.1434**	0.0000														
T1	−0.0488	−0.0878	0.1315	−0.1488	−0.1813	−0.0528	−0.1277	−0.1834	−0.0853	−0.0455	0.1294	−0.0601	−0.1153	−0.0230	−0.0005	−0.1454	−0.1488	−0.1853	−0.0555	0.0308	−0.0135	−0.0207	−0.1767	−0.0556	−0.1608	−0.0790	−0.1589	0.0611	0.0000													
T2	−0.0701	−0.0744	−0.0084	−0.0355	0.0120	−0.0744	−0.0754	−0.0243	−0.0715	−0.0886	0.0017	−0.0761	−0.0761	−0.0896	−0.0652	−0.0630	−0.0355	−0.0152	−0.0835	−0.0251	−0.0838	−0.0624	−0.0127	−0.0925	−0.0358	−0.0782	0.0211	−0.0619	−0.0435	0.0000												
C1	−0.0230	−0.0223	0.0379	0.0092	0.0397	−0.0222	−0.0230	0.0201	−0.0216	−0.0338	0.0492	−0.0238	−0.0300	−0.0343	−0.0137	−0.0124	0.0092	0.0268	−0.0305	0.0286	−0.0304	−0.0109	0.0227	−0.0378	0.0020	−0.0257	0.0323	−0.0120	−0.0627	−0.0831	0.0000											
C2	−0.0339	−0.0266	−0.0231	0.0610	**0.1226**	−0.0412	−0.0055	0.0765	0.0309	−0.0037	−0.0146	−0.0309	−0.0146	−0.0151	−0.0392	0.0226	**0.0610**	**0.0886**	−0.0250	−0.0266	−0.0312	−0.0312	0.0939	−0.0052	0.0685	−0.0270	0.1295	−0.0471	0.0411	−0.0711	−0.0202	0.0000										
C3	0.0083	0.0253	−0.0311	**0.1569**	**0.2317**	0.0154	0.0656	**0.1700**	0.1401	0.0950	−0.0315	0.0230	0.0681	**0.0781**	−0.0027	**0.1106**	**0.1569**	**0.1862**	0.0469	−0.0279	0.0409	0.0075	**0.1964**	0.0913	**0.1681**	0.0310	**0.2421**	−0.0147	0.1140	0.0044	0.0562	−0.0118	0.0000									
C4	−0.0108	−0.0195	0.0649	−0.0030	0.0217	−0.0161	−0.0267	0.0062	−0.0345	−0.0403	0.0763	−0.0193	−0.0277	−0.0372	−0.0018	−0.0217	−0.0030	0.0117	−0.0295	0.0476	−0.0268	−0.0011	0.0066	−0.0452	−0.0129	−0.0232	0.0129	0.0056	−0.0721	−0.0840	−0.0307	−0.0047	0.0868	0.0000								
V1	0.0148	−0.0014	**0.1152**	−0.0141	−0.0066	0.0086	−0.0195	−0.0123	−0.0349	−0.0270	**0.1252**	0.0035	−0.0189	−0.0174	0.0320	−0.0239	−0.0141	−0.0094	−0.0089	**0.0847**	0.0003	0.0224	−0.0158	−0.0339	−0.0300	−0.0040	−0.0177	0.0490	−0.1276	−0.0603	−0.0124	0.0364	**0.1364**	−0.0243	0.0000							
V2	0.0242	0.0063	**0.1235**	−0.0138	−0.0117	0.0185	−0.0146	−0.0181	−0.0202	−0.0089	**0.1319**	0.0138	−0.0113	0.0021	0.0431	−0.0194	−0.0138	−0.0208	0.0046	**0.0882**	0.0174	0.0377	−0.0192	−0.0172	−0.0290	0.0050	−0.0180	0.0610	−0.1723	−0.0462	0.0012	0.0478	0.1333	−0.0097	−0.0212	0.0000						
V3	−0.0292	−0.0224	0.0280	−0.0009	0.0428	−0.0232	−0.0214	0.0160	−0.0163	−0.0295	0.0341	−0.0242	−0.0276	−0.0308	−0.0167	−0.0092	0.0056	0.0234	−0.0291	0.0210	−0.0294	−0.0238	0.0255	−0.0335	−0.0046	−0.0256	0.0357	−0.0168	−0.0721	−0.0807	−0.0345	−0.0241	0.0428	−0.0271	−0.0083	0.0029	0.0000					
V4	0.0223	0.0033	**0.1334**	−0.0179	−0.0156	0.0159	−0.0179	−0.0182	−0.0369	−0.0240	**0.1426**	0.0100	−0.0172	−0.0121	0.0429	−0.0256	−0.0179	−0.0162	−0.0031	**0.0964**	0.0085	0.0374	−0.0231	−0.0316	−0.0361	0.0014	−0.0283	0.0649	−0.1360	−0.0519	−0.0075	0.0515	**0.1558**	−0.0217	−0.0315	−0.0247	−0.0032	0.0000				
M1	0.0026	0.0130	−0.0139	**0.0969**	**0.1416**	0.0055	0.0294	**0.1067**	**0.0775**	0.0496	−0.0119	0.0060	0.0384	0.0410	−0.0048	**0.0672**	**0.0969**	**0.1192**	0.0230	−0.0136	0.0200	0.0025	**0.1189**	0.0435	**0.0929**	0.0142	**0.1420**	−0.0143	−0.0121	−0.0296	0.0293	−0.0153	−0.0204	0.0463	**0.0783**	0.0755	0.0214	0.0887	0.0000			
M2	−0.0456	−0.0500	0.0160	−0.0123	0.0308	−0.0499	−0.0505	−0.0004	−0.0485	−0.0645	0.0264	−0.0517	−0.0517	−0.0654	−0.0407	−0.0389	−0.0123	0.0078	−0.0592	0.0008	−0.0597	−0.0378	0.0085	−0.0682	−0.0141	−0.0537	0.0340	−0.0374	−0.0286	−0.1163	−0.0590	−0.0463	0.0325	−0.0600	−0.0370	−0.0212	−0.0564	−0.0294	−0.0015	0.0000		
M3	0.0052	−0.0696	−0.0246	−0.0632	−0.0702	−0.0822	−0.0664	−0.0686	−0.0620	0.1075	−0.0304	−0.0606	−0.0469	0.0505	−0.0394	0.1016	−0.0664	−0.0187	−0.0720	−0.0412	−0.0254	−0.0023	−0.0757	−0.0708	−0.0877	−0.0403	−0.0860	0.0342	−0.1748	−0.0857	−0.0461	0.0195	0.1105	−0.0615	−0.0781	−0.0753	−0.0433	−0.0850	0.0335	−0.0629	0.0000	
M4	0.1088	0.0313	0.0656	0.0091	−0.0154	0.0915	0.0175	−0.0108	0.1063	**0.2116**	0.0610	−0.0039	0.1189	0.1244	0.0333	0.2149	0.0175	0.0480	0.0058	0.0755	0.1127	0.0908	0.0183	0.0975	0.0329	0.0458	0.0972	0.1611	−0.1321	0.0763	0.0442	0.1449	0.1970	0.0764	0.0423	−0.0121	0.0318	0.0477	0.0862	0.0994	0.0198	0.0000

**Table 3:** Numbers of effective migrants per generation (*N*_*e*_*m*) in the *Sogatella furcifera* populations found in the Greater Mekong Subregion (GMS).

Population, *i*	*ϴi*	JP → *i*	KY → *i*	YS → *i*	FN → *i*	MD → *i*	BS → *i*	MS → *i*	YIJ → *i*	LC → *i*	NE → *i*	MH → *i*	SJ → *i*	GM → *i*	CY → *i*	CX → *i*	SM → *i*	XP → *i*	YUJ → *i*	ZY → *i*	SZ → *i*	L1 → *i*	L2 → *i*	L3 → *i*	L4 → *i*	L5 → *i*	L6 → *i*	L7 → *i*	L8 → *i*	T1 → *i*	T2 → *i*	C1→*i*	C2 → *i*	C3 → *i*	C4 → *i*	V1 → *i*	V2 → *i*	V3 → *i*	V4 → *i*	M1 → *i*	M2 → *i*	M3 → *i*	M4 → *i*	Total *i*
JP	0.08934	—	66.7	74.4	62.1	36.3	71.1	60.8	33.3	77.7	56.3	13.5	81.7	36.1	62.1	80.4	17.6	79.3	54.2	16.1	29.5	11.8	36.2	30.7	31.2	74.1	77.2	21.8	55.1	80.3	56.2	9.0	38.2	24.9	56.5	80.8	19.0	21.5	7.2	74.7	39.0	63.4	8.0	1925.8
KY	0.00002	0.0	—	0.0	0.0	0.0	0.0	0.0	0.0	0.0	0.0	0.0	0.0	0.0	0.0	0.0	0.0	0.0	0.0	0.0	0.0	0.0	0.0	0.0	0.0	0.0	0.0	0.0	0.0	0.0	0.0	0.0	0.0	0.0	0.0	0.0	0.0	0.0	0.0	0.0	0.0	0.0	0.0	0.4
YS	0.06539	28.1	58.8	—	5.9	37.2	60.6	57.9	52.0	13.9	52.1	51.3	45.2	46.8	26.5	55.7	49.9	57.6	36.9	17.4	21.5	12.7	13.5	33.3	58.8	13.6	12.3	8.4	39.1	52.4	30.6	20.2	24.7	14.4	42.1	32.0	49.0	32.7	11.3	50.6	41.0	8.5	10.9	1387.4
FN	0.00003	0.0	0.0	0.0	—	0.0	0.0	0.0	0.0	0.0	0.0	0.0	0.0	0.0	0.0	0.0	0.0	0.0	0.0	0.0	0.0	0.0	0.0	0.0	0.0	0.0	0.0	0.0	0.0	0.0	0.0	0.0	0.0	0.0	0.0	0.0	0.0	0.0	0.0	0.0	0.0	0.0	0.0	0.7
MD	0.00005	0.0	0.0	0.0	0.0	—	0.0	0.0	0.0	0.0	0.0	0.0	0.0	0.0	0.0	0.0	0.0	0.0	0.0	0.0	0.0	0.0	0.0	0.0	0.0	0.0	0.0	0.0	0.0	0.0	0.0	0.0	0.0	0.0	0.0	0.0	0.0	0.0	0.0	0.0	0.0	0.0	0.0	1.2
BS	0.00007	0.0	0.1	0.1	0.0	0.0	—	0.0	0.0	0.1	0.1	0.1	0.1	0.1	0.1	0.1	0.0	0.0	0.1	0.1	0.1	0.0	0.1	0.0	0.0	0.1	0.1	0.0	0.0	0.0	0.1	0.1	0.0	0.1	0.1	0.1	0.0	0.1	0.1	0.1	0.1	0.0	0.0	1.8
MS	0.01343	12.5	8.4	1.8	12.2	2.1	10.8	—	9.1	11.9	11.4	3.4	12.1	4.8	2.7	4.8	5.7	1.1	9.5	11.2	9.6	4.2	12.4	9.6	2.6	10.4	12.2	4.8	8.7	1.7	10.8	4.1	3.7	3.2	6.6	7.7	12.6	6.6	7.0	11.1	10.9	11.9	12.4	320.3
YIJ	0.00001	0.0	0.0	0.0	0.0	0.0	0.0	0.0	—	0.0	0.0	0.0	0.0	0.0	0.0	0.0	0.0	0.0	0.0	0.0	0.0	0.0	0.0	0.0	0.0	0.0	0.0	0.0	0.0	0.0	0.0	0.0	0.0	0.0	0.0	0.0	0.0	0.0	0.0	0.0	0.0	0.0	0.0	0.3
LC	0.00004	0.0	0.0	0.0	0.0	0.0	0.0	0.0	0.0	—	0.0	0.0	0.0	0.0	0.0	0.0	0.0	0.0	0.0	0.0	0.0	0.0	0.0	0.0	0.0	0.0	0.0	0.0	0.0	0.0	0.0	0.0	0.0	0.0	0.0	0.0	0.0	0.0	0.0	0.0	0.0	0.0	0.0	0.9
NE	0.00002	0.0	0.0	0.0	0.0	0.0	0.0	0.0	0.0	0.0	—	0.0	0.0	0.0	0.0	0.0	0.0	0.0	0.0	0.0	0.0	0.0	0.0	0.0	0.0	0.0	0.0	0.0	0.0	0.0	0.0	0.0	0.0	0.0	0.0	0.0	0.0	0.0	0.0	0.0	0.0	0.0	0.0	0.4
MH	0.09457	27.7	69.2	55.3	64.2	18.6	83.9	47.0	67.3	41.4	72.8	—	78.5	56.3	43.0	83.6	86.0	87.0	76.6	8.4	34.5	7.9	68.6	79.1	8.7	27.3	70.9	66.9	7.8	73.3	65.7	75.8	12.5	41.6	17.6	39.3	38.2	22.5	56.0	10.1	23.5	35.0	61.7	2011.3
SJ	0.03453	5.2	8.9	13.2	11.6	18.3	23.5	12.3	21.1	20.1	28.2	26.5	—	14.6	5.5	17.0	28.9	6.6	2.1	3.3	12.8	1.9	4.6	25.8	13.7	9.5	23.9	5.7	32.3	29.1	3.0	10.9	9.3	5.2	20.9	7.3	6.1	5.5	3.2	2.3	6.0	11.5	6.8	524.1
GM	0.00003	0.0	0.0	0.0	0.0	0.0	0.0	0.0	0.0	0.0	0.0	0.0	0.0	—	0.0	0.0	0.0	0.0	0.0	0.0	0.0	0.0	0.0	0.0	0.0	0.0	0.0	0.0	0.0	0.0	0.0	0.0	0.0	0.0	0.0	0.0	0.0	0.0	0.0	0.0	0.0	0.0	0.0	0.7
CY	0.00256	1.7	1.1	1.0	1.7	2.1	1.7	2.2	0.4	1.3	0.8	1.0	1.0	1.8	—	0.4	1.5	2.1	1.9	1.0	0.6	2.1	1.0	1.0	0.5	0.6	0.7	1.4	0.9	1.6	0.4	1.3	0.3	1.4	0.4	2.2	1.4	0.3	0.4	0.6	0.4	0.7	0.6	45.6
CX	0.06575	60.4	53.0	56.9	44.2	51.5	59.5	21.7	23.9	55.7	57.9	59.8	21.4	47.9	55.7	—	36.6	12.2	58.4	47.6	28.9	49.6	3.8	18.0	62.5	16.3	58.3	19.0	41.9	39.2	19.9	59.4	54.4	36.7	30.0	48.6	15.5	56.2	60.5	18.4	60.2	53.1	51.9	1726.4
SM	0.00006	0.0	0.0	0.0	0.0	0.0	0.0	0.0	0.0	0.0	0.1	0.0	0.0	0.0	0.0	0.0	—	0.0	0.0	0.0	0.0	0.0	0.0	0.1	0.0	0.1	0.1	0.0	0.0	0.0	0.0	0.0	0.0	0.0	0.0	0.0	0.0	0.0	0.0	0.1	0.0	0.0	0.0	1.3
XP	0.01418	11.7	1.0	7.7	12.5	12.6	8.5	13.2	10.4	12.8	4.6	11.5	1.1	10.6	5.8	7.2	11.8	—	11.0	3.9	2.6	5.3	12.5	4.5	6.1	7.6	12.2	12.4	10.2	8.3	12.6	7.7	3.8	7.1	5.3	5.9	10.2	4.2	11.9	13.0	9.8	7.1	6.5	344.9
YUJ	0.06249	51.5	44.9	56.2	33.4	40.1	42.2	13.0	8.5	53.0	42.7	54.3	45.8	41.6	48.1	50.8	26.2	40.5	—	36.4	20.0	5.1	38.6	53.7	9.7	23.9	47.3	20.1	57.3	47.6	57.8	54.6	10.5	37.5	28.1	27.9	34.9	27.8	13.5	44.3	47.9	14.6	41.6	1493.6
ZY	0.03726	31.1	30.7	11.8	29.6	18.2	28.6	25.9	27.6	18.1	32.0	21.5	4.3	31.2	21.4	12.0	22.4	27.2	11.0	—	34.6	21.3	19.3	4.2	34.2	32.4	4.9	6.9	32.5	32.6	20.5	33.9	20.3	6.6	17.5	15.2	20.9	32.9	25.6	27.2	14.5	7.3	12.6	882.8
SZ	0.01998	12.5	7.9	3.4	10.7	4.5	14.3	14.6	9.9	6.2	10.9	2.9	9.1	17.5	17.0	2.8	1.2	2.1	5.3	10.5	—	13.3	4.5	16.5	9.8	5.8	17.5	8.9	2.2	8.4	5.0	14.7	10.5	2.0	8.2	6.0	16.5	3.3	15.4	6.6	1.6	6.5	12.8	359.5
L1	0.00023	0.1	0.0	0.2	0.1	0.0	0.1	0.2	0.0	0.2	0.2	0.2	0.1	0.2	0.1	0.1	0.1	0.2	0.2	0.2	0.1	—	0.0	0.2	0.1	0.2	0.1	0.1	0.1	0.2	0.1	0.1	0.1	0.2	0.2	0.0	0.2	0.0	0.1	0.1	0.1	0.2	0.1	4.8
L2	0.03014	19.3	19.6	26.0	6.9	25.5	17.6	19.2	22.1	26.1	23.6	27.4	4.3	22.3	18.5	24.6	27.7	9.7	16.7	24.2	22.1	14.2	—	13.4	25.7	23.7	22.5	15.1	25.8	5.7	24.3	26.2	18.0	17.6	9.2	26.7	23.0	2.6	11.7	20.2	20.6	25.9	26.9	802.4
L3	0.00001	0.0	0.0	0.0	0.0	0.0	0.0	0.0	0.0	0.0	0.0	0.0	0.0	0.0	0.0	0.0	0.0	0.0	0.0	0.0	0.0	0.0	0.0	—	0.0	0.0	0.0	0.0	0.0	0.0	0.0	0.0	0.0	0.0	0.0	0.0	0.0	0.0	0.0	0.0	0.0	0.0	0.0	0.2
L4	0.00000	0.0	0.0	0.0	0.0	0.0	0.0	0.0	0.0	0.0	0.0	0.0	0.0	0.0	0.0	0.0	0.0	0.0	0.0	0.0	0.0	0.0	0.0	0.0	—	0.0	0.0	0.0	0.0	0.0	0.0	0.0	0.0	0.0	0.0	0.0	0.0	0.0	0.0	0.0	0.0	0.0	0.0	0.0
L5	0.09400	69.4	20.9	49.7	66.4	22.8	78.7	77.7	35.2	44.4	70.0	31.1	69.3	29.6	13.1	44.4	82.3	74.8	50.3	55.7	75.4	72.1	68.9	39.4	81.2	—	37.8	45.0	63.1	41.7	24.0	12.6	11.7	70.8	63.6	15.1	10.2	54.5	82.5	8.8	16.4	17.9	77.7	1976.2
L6	0.06442	27.2	19.8	12.9	12.3	18.9	13.6	47.2	13.8	12.3	29.2	24.3	22.0	10.1	27.1	20.2	8.0	15.0	48.5	24.9	4.8	27.4	4.7	16.2	5.5	13.8	—	48.1	26.6	10.3	6.1	57.2	8.8	58.1	28.2	13.0	8.2	12.6	30.2	41.4	8.1	43.1	20.9	900.3
L7	0.00001	0.0	0.0	0.0	0.0	0.0	0.0	0.0	0.0	0.0	0.0	0.0	0.0	0.0	0.0	0.0	0.0	0.0	0.0	0.0	0.0	0.0	0.0	0.0	0.0	0.0	0.0	—	0.0	0.0	0.0	0.0	0.0	0.0	0.0	0.0	0.0	0.0	0.0	0.0	0.0	0.0	0.0	0.2
L8	0.02505	21.4	23.2	4.7	22.8	2.9	16.9	16.1	10.6	19.6	15.3	20.3	13.2	22.2	15.0	21.0	18.7	20.7	13.8	17.9	3.9	23.7	23.2	7.0	12.0	17.9	19.5	4.0	—	2.1	6.0	7.4	19.8	20.4	7.2	14.0	9.4	1.5	7.2	10.3	5.6	9.8	12.3	560.6
T1	0.04210	31.5	25.1	26.4	34.3	33.9	10.0	4.6	18.1	11.9	2.3	5.5	5.7	30.2	29.6	20.4	3.2	25.9	30.9	36.7	7.3	16.4	9.4	8.8	29.5	3.9	30.9	10.3	30.8	—	14.0	13.2	20.9	6.0	10.1	4.8	15.4	3.8	16.0	9.8	2.0	9.9	36.2	695.2
T2	0.03474	13.3	4.7	11.5	6.3	10.1	16.3	2.6	19.7	13.3	27.5	16.3	27.9	6.0	11.1	10.3	12.7	10.8	17.2	12.9	3.9	22.3	12.3	14.2	10.4	20.3	12.9	11.5	15.7	28.3	-	19.1	28.9	30.1	21.6	18.3	27.2	28.7	31.1	30.7	26.9	28.9	9.2	702.9
C1	0.06384	9.6	54.0	48.4	32.2	31.0	9.8	48.4	39.8	56.0	25.4	43.1	47.7	21.5	56.4	27.5	14.1	29.6	38.3	10.8	46.2	52.0	8.8	56.0	7.7	36.4	50.3	38.5	28.1	17.4	23.4	—	6.3	21.3	43.5	19.3	14.1	23.9	50.7	42.0	18.9	9.8	40.1	1298.0
C2	0.08735	60.9	52.3	19.6	13.1	57.6	10.7	75.3	55.9	18.1	8.3	22.7	9.3	15.9	37.3	14.6	14.1	36.3	26.5	76.3	28.5	44.5	17.0	22.1	52.2	47.6	42.3	13.4	64.9	10.2	14.4	49.5	—	42.9	39.5	57.6	13.9	75.1	35.5	12.5	34.2	33.6	70.6	1446.7
C3	0.08389	6.8	54.4	41.9	64.1	8.9	48.8	48.5	72.6	10.8	8.6	18.4	25.0	23.7	21.9	10.8	24.7	64.8	44.6	57.2	49.0	69.1	74.3	25.9	17.2	8.4	35.2	20.8	62.0	13.4	48.8	12.0	9.2	—	17.8	22.8	21.4	8.0	38.3	57.2	27.8	35.8	21.2	1351.9
C4	0.00002	0.0	0.0	0.0	0.0	0.0	0.0	0.0	0.0	0.0	0.0	0.0	0.0	0.0	0.0	0.0	0.0	0.0	0.0	0.0	0.0	0.0	0.0	0.0	0.0	0.0	0.0	0.0	0.0	0.0	0.0	0.0	0.0	0.0	—	0.0	0.0	0.0	0.0	0.0	0.0	0.0	0.0	0.3
V1	0.00002	0.0	0.0	0.0	0.0	0.0	0.0	0.0	0.0	0.0	0.0	0.0	0.0	0.0	0.0	0.0	0.0	0.0	0.0	0.0	0.0	0.0	0.0	0.0	0.0	0.0	0.0	0.0	0.0	0.0	0.0	0.0	0.0	0.0	0.0	—	0.0	0.0	0.0	0.0	0.0	0.0	0.0	0.5
V2	0.06373	24.4	14.7	25.4	56.1	58.5	50.9	57.5	8.6	54.5	57.4	55.4	54.7	17.2	31.5	47.0	56.9	16.7	42.4	42.6	29.6	29.8	36.7	8.6	54.3	48.2	39.0	38.6	29.3	31.4	55.6	49.3	26.8	46.6	27.0	48.0	-	21.6	58.1	43.4	49.4	16.8	41.0	1601.5
V3	0.02542	5.8	18.8	21.4	22.3	21.6	19.7	14.6	17.4	13.2	23.8	21.4	5.6	11.3	10.3	4.3	20.3	16.7	5.4	22.7	11.0	18.5	15.2	15.4	13.5	22.9	9.0	13.4	2.3	14.2	13.5	13.7	19.5	5.9	17.7	22.4	18.9	—	5.4	16.3	6.9	1.3	7.5	581.0
V4	0.02338	6.0	16.2	5.0	2.1	7.2	21.1	3.9	5.8	11.1	1.7	11.2	19.4	21.1	19.0	8.2	20.6	21.5	17.3	7.5	6.2	2.1	12.4	3.9	13.4	2.1	1.9	3.8	1.3	11.6	4.4	2.8	8.3	1.5	6.2	19.8	5.9	4.4	—	10.3	4.4	17.6	4.0	374.3
M1	0.03383	25.5	12.0	4.5	9.5	4.0	12.2	12.4	14.0	9.3	9.5	23.4	16.7	18.2	13.0	5.5	20.3	24.4	26.4	23.6	4.9	24.0	1.8	9.6	22.7	28.9	2.8	21.1	4.9	5.5	19.0	4.4	14.6	19.4	29.3	16.2	5.3	10.6	5.4	—	14.4	11.5	17.3	578.0
M2	0.00002	0.0	0.0	0.0	0.0	0.0	0.0	0.0	0.0	0.0	0.0	0.0	0.0	0.0	0.0	0.0	0.0	0.0	0.0	0.0	0.0	0.0	0.0	0.0	0.0	0.0	0.0	0.0	0.0	0.0	0.0	0.0	0.0	0.0	0.0	0.0	0.0	0.0	0.0	0.0	—	0.0	0.0	0.3
M3	0.03151	9.8	27.2	13.3	28.1	19.9	23.8	7.5	23.3	21.8	25.9	20.5	25.7	10.2	22.4	27.6	25.7	27.5	27.0	25.8	24.3	10.1	24.4	18.1	9.2	3.4	17.5	5.9	27.1	13.1	22.1	11.9	22.7	28.3	20.2	26.9	21.7	23.8	16.9	20.1	26.4	—	8.4	815.7
M4	0.01172	1.2	4.3	7.8	4.2	4.3	2.3	3.7	7.5	1.9	2.3	6.9	8.0	10.8	5.7	6.3	9.3	2.0	3.7	3.3	1.1	6.4	8.4	0.9	1.0	9.1	2.3	9.3	10.0	1.8	1.1	1.7	10.1	9.9	3.9	5.9	5.5	7.2	2.8	4.3	10.0	1.1	—	209.2

**Table 4:** Population data of *Sogatella furcifera* populations in the Greater Mekong Subregion (GMS) during 2014–2015.

Regions	Code	Location	Longitude	Latitude	Elevation (meter)	Date*
South Yunnan, China	JP	Jinping, Yunnan	N22.8	E103.2	1349	June 10
	KY	Kaiyuan, Yunnan	N23.5	E103.3	1305.9	June 10
Southeast Yunnan, China	YS	Yanshan, Yunnan	N23.6	E104.3	1579	June 9
	FN	Funing, Yunnan	N23.6	E105.6	680	June 10
West Yunnan, China	MD	Midu, Yunnan	N25.3	E100.4	1653	June 27
	BS	Baoshan, Yunnan	N25.0	E99.1	1699.9	June 26
	MS	Mangshi, Yunnan	N24.3	E98.4	851.2	June 27
	YIJ	Yingjiang, Yunnan	N24.7	E97.9	1682	June 30
	LC	LongChuan, Yunnan	N24.1	E97.7	953	May 27
Southwest Yunnan, China	NE	Ninger, Yunnan	N23.0	E101.0	1312.9	June 16
	MH	Menghai, Yunnan	N21.9	E100.4	1230	May 15
	SJ	Shuangjiang, Yunnan	N23.4	E99.8	1063	July 10
	GM	Gengma, Yunnan	N23.5	E99.3	1116	July 10
	CY	Cangyuan, Yunnan	N23.1	E99.2	1444	June 4
Central Yunnan, China	CX	Chuxiong, Yunnan	N25.0	E101.4	1812.8	June 26
	SM	Songming, Yunnan	N25.3	E103.0	1875.9	July 3
	XP	Xinping, Yunnan	N24.0	E101.9	1502.2	June 17
	YUJ	Yuanjiang, Yunnan	N23.7	E102.0	1202.7	June 17
Northeast Yunnan, China	ZY	Zhaoyang, Yunnan	N27.3	E103.7	1907	July 10
	SZ	Shizong, Yunnan	N24.6	E1042.9	951	June 14
Laos	L1	Hadsayphong District, Vientiane Capital City	N18.2	E102.5	128	March 18
	L2	Thaphabad District, Bolikhamxay Province	N18.4	E103.2	128	March 19
	L3	Bolikhan District, Bolikhamxay Province	N18.3	E103.6	128	March 19
	L4	Hinboun District, Khammouane Province	N17.7	E104.5	130	March 20
	L5	Vapee District, Saravanh Province	N15.6	E105.9	120	March 21
	L6	Saravanh District, Saravanh Province	N15.6	E106.3	223	March 21
	L7	Vapee District, Saravan Province	N15.6	E105.9	119	March 21
	L8	Songkhone District, Savonakhet Province	N16.2	E105.2	115	March 22
Thailand	T1	Nakhon Chum District, Kamphaeng Phet Province	N16.4	E99.4	53	May 14
	T2	Bang Len district, Nakhon Pathom Province	N14.0	E100.2	−11	May 15
Cambodia	C1	Sangkat Prateahlang, Khan Dangkor, Phnom penh	N11.4	E103.2	14	March 24
	C2	Sangkat Dangkor, Khan Dangkor, Phnom penh	N11.5	E104.9	12	March 24
	C3	Stoung District, Kampoug Thom Province	N13.0	E104.5	10	March 27
	C4	Aek Phnum District Battambang Province	N13.3	E103.6	7	March 27
Vietnam	V1	Xuan Linh Commune, Nghi Xuan District Ha Tinh Province	N18.5	E105.7	10	April 16
	V2	Quang Ninh District, Quang Binh Province	N17.4	E106.6	10	April 17
	V3	Phong An Commune, Phong Dien District, Hue Province	N16.5	E107.3	4.8	April 17
	V4	Phu Loc District, Hue City	N16.3	E107.7	1.4	April 18
Myanmar	M1	Begayet, Ayeyarwady region	N16.8	E94.8	5.5	August 18
	M2	Pathwe, Ayeyarwady region	N17.0	E95.2	2.8	August 19
	M3	Kali, Bago region	N17.3	E96.5	25	August 20
	M4	Kanbaukkyi, Bago region	N18.9	E96.3	60	August 20

^*^All samples were collected in 2014 except those in Myanmar were collected in 2015.
