# Supplementary material for: Possible Source Populations of the White-backed Planthopper in the Greater Mekong Subregion Revealed by Mitochondrial DNA Analysis
Source: Sci Rep. 2016 Dec 19;6:39167. doi: 10.1038/srep39167 (PMC5171772; doi:10.1038/srep39167)
Supplement: Supplementary Legends [file srep39167-s1.pdf]

SUPPLEMENTARY LEGENDS

Possible Source Populations of the White-backed Planthopper in the Greater Mekong Subregion Revealed by Mitochondrial DNA Analysis

Xiang-yong Li[1*], Dong Chu[2*], Yan-qiong Yin[1], Xue-qing Zhao[1], Ai-dong Chen[1], Khay Sathya[3], Bounneuang Douangboupha[4], Mu Mu Kyaw[5], Manita Kongchuensin[6], Ngo Vinh Vien[7], Nguyen Huy Chung[7]**

[1] Agriculture Environment and Resources Institute, Yunnan Academy of Agricultural Sciences, Kunming 650205, China

[2] Key Lab of Integrated Crop Pest Management of Shandong Province, College of Agronomy and Plant Protection, Qingdao Agricultural University, Qingdao 266109, China

[3] Plant Protection Office, Cambodian Agricultural Research and Development Institute, Phnom Penh 01, Cambodia

[4] Horticulture Research Center, National Agriculture and Forestry Research Institute, Vientiane 7170, Lao PDR

[5] Myanmar Academy of Agricultural, Forestry, Livestock, and Fishery Sciences, Nay Pyi Taw, Myanmar

[6] Plant Protection Research and Development Office, Department of Agriculture, Bangkok 10170, Thailand

[7] Plant Protection Research Institute, Vietnam Academy of Agricultural Sciences, Hanoi, Vietnam

* These authors contributed to this work equally.

**To whom correspondence should be addressed: Ai-dong Chen, Email: shenad68@163.com

Table S1 Distribution of mitochondrial COI haplotypes of *Sogatella furcifera* populations in the Greater Mekong Subregion (GMS)

	CX	MD	BS	MS	YIJ	LC	XP	YUJ	NE	MH	SJ	GM	CY	SZ	KY	XS	FN	JP	SM	SY	L1	L2	L3	L4	L5	L6	L7	L8	T1	T2	C1	C2	C3	C4	V1	V2	V3	V4	M1	M2	M3	M4
Hap1	14	21	13	11	16	19	18	17	18	11	14	14	19	11	15	12	17	15	16	16	18	14	18	15	14	14	20	11	3	6	18	10	8	17	17	11	15	18	6	9	7	4
Hap2								1																																		
Hap3	6	2	7	4	2	4	2	2	5	12	6	5	6	7	5	10	3	5	4	5	6	7	2	4	2	4	2	7		2	5	6	6	5	3	2	5	2	5	3	1	
Hap4								1																																		
Hap5								1								1																										
Hap6							1	1									2	1				2			1												1					
Hap7					1																																					
Hap8					1																																					
Hap9					1																																					
Hap10																																						1				
Hap11																																					1					
Hap12																		1																			1					
Hap13																																				1						
Hap14																																				1						
Hap15																																				1						
Hap16																																			1							
Hap17																																			1							
Hap18																1																										
Hap19							1																																			
Hap20							1				1																															
Hap21														1																												
Hap22														1																												
Hap23														1																												

Hap24					1									
Hap25					1									
Hap26					1									
Hap27								1						
Hap28						1		1						
Hap29							1							
Hap30		1					1							
Hap31							1							
Hap32							1							
Hap33				1										
Hap34	1	1		1										1
Hap35		1												
Hap36		1												
Hap37			1											
Hap38	1													
Hap39													1	
Hap40												1		
Hap41												1		
Hap42												1		
Hap43												1		
Hap44											1			
Hap45										1				
Hap46									1					
Hap47						1								
Hap48						1								
Hap49						1								

Hap50						1								
Hap51								1						
Hap52								1						
Hap53							1	1						
Hap54												1		
Hap55											1			
Hap56											1			
Hap57		1			1					1				
Hap58									1					
Hap59					1									
Hap60					1									
Hap61					1									1
Hap62				1										
Hap63	1													
Hap64	1													
Hap65	1													
Hap66		1												
Hap67		1												
Hap68			1											
Hap69													1	
Hap70													1	
Hap71													1	
Hap72													1	
Hap73													2	

Table S2 Unidirectional estimates of M of *Sogatella furcifera* populations in the Greater Mekong Subregion (GMS)

Population, *i*	Θi	JP→*i*	KY→*i*	YS→*i*	FN→*i*	MD→*i*	BS→*i*	MS→*i*	YIJ→*i*	LC→*i*	NE→*i*	MH→*i*	SJ→*i*
JP	0.08934	-	746.1	832.6	694.7	405.8	795.3	680.8	373.3	869.4	630.0	150.6	914.6
KY	0.00002	286.1	-	708.2	630.3	143.2	406.3	140.3	145.6	357.7	781.7	150.8	75.2
YS	0.06539	430.3	898.8	-	90.4	568.9	926.8	886.2	795.5	212.5	797.3	783.9	690.9
FN	0.00003	779.6	787.7	243.9	-	477.0	912.7	362.6	556.8	226.7	457.5	569.6	682.0
MD	0.00005	757.5	500.6	99.6	818.3	-	723.6	183.4	353.7	372.3	564.6	402.0	646.8
BS	0.00007	181.2	814.8	886.3	317.8	290.7	-	479.8	415.8	813.1	897.4	803.9	733.1
MS	0.01343	933.5	629.1	133.5	905.7	153.6	804.3	-	680.8	885.2	851.4	249.7	899.8
YIJ	0.00001	557.5	505.7	350.5	578.2	226.8	796.6	874.0	-	925.6	769.5	811.8	207.9
LC	0.00004	895.0	915.8	373.5	883.4	738.3	716.0	128.2	725.5	-	645.8	95.0	775.3
NE	0.00002	679.9	289.1	572.0	583.5	290.9	97.0	527.2	187.1	664.1	-	883.9	51.5
MH	0.09457	292.9	731.4	584.2	678.8	196.2	887.5	496.7	711.9	437.5	770.0	-	830.4
SJ	0.03453	149.2	257.7	382.2	334.5	530.5	680.4	357.6	610.4	582.1	816.9	768.4	-
GM	0.00003	831.2	585.8	419.4	601.8	87.8	318.8	850.9	110.4	764.5	300.8	150.5	116.0
CY	0.00256	644.8	444.5	396.6	666.2	805.0	681.2	871.9	162.0	510.2	323.1	407.8	393.3
CX	0.06575	919.2	805.7	864.8	671.7	782.7	905.1	330.7	362.8	846.4	880.5	909.0	326.1
SM	0.00006	72.7	339.4	807.2	97.2	664.2	552.9	230.5	161.2	209.8	898.2	742.4	103.9
XP	0.01418	825.6	67.6	544.0	884.2	890.0	600.8	928.4	735.4	902.9	321.9	810.6	81.0
YUJ	0.06249	824.7	718.7	899.8	534.6	640.9	676.1	208.0	136.3	848.8	683.3	869.1	733.1
SY	0.03726	834.9	823.8	317.8	795.6	487.6	766.5	695.0	740.3	486.8	858.4	576.6	116.3
SZ	0.01998	626.6	397.5	168.5	535.7	226.1	717.5	731.7	493.0	312.7	545.6	143.1	457.9
L1	0.00023	420.8	140.7	699.6	511.8	161.4	398.9	804.4	154.6	800.4	885.7	726.1	407.8
L2	0.03014	641.5	649.3	863.3	227.7	844.6	584.8	638.4	734.8	864.6	781.8	907.9	144.1
L3	0.00001	148.9	781.7	180.5	131.4	648.0	321.0	682.5	723.3	644.2	419.2	247.4	496.6
L4	0.00000	912.4	825.0	667.5	402.9	933.2	630.0	349.6	405.4	784.5	172.8	790.6	848.0
L5	0.09400	738.7	222.0	528.2	705.9	242.5	837.6	826.7	374.2	472.1	744.6	331.1	737.2
L6	0.06442	421.7	307.1	199.5	190.2	292.8	210.6	731.9	215.0	190.7	453.0	376.8	341.2
L7	0.00001	289.8	288.7	133.4	573.5	627.6	122.6	418.6	484.9	181.7	193.2	717.0	221.8
L8	0.02505	854.1	928.1	189.4	910.2	114.1	674.0	642.4	423.7	780.9	611.5	812.0	527.1
T1	0.04210	747.1	596.2	626.1	814.6	804.2	237.6	108.5	429.8	282.5	54.3	130.3	135.0
T2	0.03474	382.4	136.0	331.5	182.8	290.5	468.1	75.3	567.9	381.9	792.7	470.0	803.6
C1	0.06384	149.8	845.2	758.2	503.9	485.3	152.8	758.8	622.7	877.4	397.9	675.3	746.6
C2	0.08735	697.4	599.2	224.2	149.6	658.9	122.9	862.4	639.6	207.5	95.2	260.3	106.4
C3	0.08389	81.6	648.4	499.2	764.0	105.8	581.2	577.8	865.1	128.9	102.2	219.5	297.9
C4	0.00002	448.4	702.8	190.1	377.8	568.7	72.2	693.3	187.3	539.2	55.9	160.7	114.4
V1	0.00002	241.0	284.0	833.6	879.1	773.7	531.5	758.9	703.1	594.7	266.5	729.2	711.8
V2	0.06373	383.0	230.6	397.9	880.5	918.3	798.4	902.5	135.7	855.7	900.1	869.8	858.3
V3	0.02542	229.2	741.1	841.3	877.5	848.8	774.9	574.9	683.8	517.8	935.7	842.4	219.7
V4	0.02338	254.7	694.5	214.1	90.2	309.6	903.1	166.9	246.5	473.8	73.8	480.5	829.0
M1	0.03383	754.4	354.0	132.9	280.3	119.2	361.8	367.0	412.7	276.3	280.9	690.6	492.2
M2	0.00002	709.9	105.1	98.1	409.1	748.8	258.2	420.7	94.6	98.3	287.5	256.7	344.2
M3	0.03151	311.4	862.9	423.5	892.6	631.5	755.3	238.7	740.8	690.7	823.0	649.3	816.5
M4	0.01172	102.6	369.8	663.3	362.4	370.4	192.2	315.2	643.9	159.5	193.0	585.5	680.0

Θ: mutation-scaled population size, which is effective population size × mutation rate per site per generation divided by the mutation rate; M: mutation-scaled immigration rate, which is the immigration rate

Table S2 Unidirectional estimates of M of Sogatella furcifera populations in the Greater Mekong Subregion (GMS) (Continued)

Population, i	GM→i	CY→i	CX→i	SM→i	XP→i	YUJ→i	SY→i	SZ→i	L1→i	L2→i	L3→i	L4→i	L5→i
JP	404.4	694.9	899.7	197.3	887.8	606.9	180.1	330.6	131.9	405.1	344.1	349.4	829.7
KY	732.1	374.3	484.3	318.2	97.9	437.6	735.2	507.0	851.7	686.7	437.1	939.2	234.2
YS	715.6	404.7	851.8	763.4	880.4	564.8	265.3	328.7	194.0	206.8	508.5	899.8	208.1
FN	618.6	309.2	342.3	903.5	321.9	105.2	96.3	348.2	435.7	537.0	476.2	248.1	222.6
MD	824.9	269.3	670.6	567.3	146.8	877.3	498.9	679.0	818.0	701.6	904.6	902.9	673.6
BS	769.9	861.2	838.0	426.3	519.7	808.5	910.8	892.3	465.7	893.6	219.3	218.1	895.9
MS	357.4	201.9	358.0	425.9	81.5	705.0	834.4	712.6	311.8	926.5	712.8	195.4	777.9
YIJ	901.7	914.4	843.5	631.0	799.6	874.7	940.5	272.9	859.0	738.5	847.9	785.9	504.9
LC	528.2	469.2	416.0	107.0	829.3	689.2	211.3	59.8	918.5	138.8	864.7	741.5	297.8
NE	170.8	419.8	448.2	530.4	690.6	301.9	651.9	156.3	133.6	744.6	933.0	332.8	494.2
MH	595.0	454.2	883.9	909.9	920.1	810.3	88.7	364.9	84.0	725.2	836.4	92.4	289.0
SJ	422.4	159.4	492.3	836.8	190.5	61.9	95.1	370.9	55.3	132.7	747.8	395.5	274.4
GM	-	541.0	779.2	737.2	751.1	156.1	901.0	633.3	874.2	902.6	532.1	460.5	928.0
CY	718.6	-	140.6	587.4	808.7	742.8	406.0	231.4	823.5	396.2	385.7	199.8	215.2
CX	729.1	846.7	-	555.9	185.1	888.1	723.5	439.4	754.4	57.7	274.1	950.9	248.2
SM	69.4	171.2	579.0	-	77.2	806.2	438.5	171.2	250.9	747.8	880.0	743.5	892.2
XP	750.1	410.6	508.9	829.3	-	772.5	275.1	185.4	376.1	881.0	320.5	428.2	533.2
YUJ	666.5	769.6	813.4	419.9	648.0	-	583.0	319.4	81.3	617.7	859.6	155.5	382.9
SY	837.7	575.6	322.7	602.1	729.4	296.2	-	929.8	570.3	518.8	112.9	918.1	870.4
SZ	875.6	852.5	140.3	58.3	105.4	266.4	526.6	-	667.9	223.5	827.4	490.9	289.9
L1	709.8	376.5	229.9	290.6	743.8	874.7	661.9	268.3	-	62.6	777.7	530.1	836.1
L2	738.7	614.7	815.6	918.9	321.2	555.2	801.8	732.9	471.0	-	444.9	853.7	785.3
L3	653.4	307.3	141.6	698.7	635.3	878.2	197.6	104.2	798.1	651.1	-	885.9	519.4
L4	779.2	697.2	808.1	127.8	742.4	187.5	534.0	155.4	546.1	600.9	464.5	-	838.1
L5	314.8	139.4	472.8	875.1	796.1	535.0	592.7	802.6	766.7	733.3	419.1	864.2	-
L6	157.1	419.9	313.2	124.6	233.1	752.5	387.2	74.2	425.1	73.3	250.8	85.8	214.5
L7	211.1	209.4	495.3	614.1	571.0	344.6	181.4	820.1	82.2	467.0	497.0	377.3	878.8
L8	887.8	599.0	839.5	745.9	827.2	549.0	712.6	156.5	946.8	926.3	280.5	479.7	713.8
T1	716.9	703.9	484.3	76.4	614.9	733.7	871.1	174.4	389.4	223.2	209.9	700.0	91.9
T2	171.6	320.8	297.4	364.4	311.0	496.1	370.2	112.6	640.9	353.2	408.4	300.0	584.4
C1	337.2	883.0	431.4	220.1	463.4	600.5	168.7	723.9	814.4	138.1	877.8	120.1	569.8
C2	181.6	427.6	166.9	161.5	415.7	302.9	873.7	325.9	509.9	194.6	253.2	597.2	544.4
C3	283.1	261.6	128.4	294.3	772.5	531.7	681.7	583.9	823.2	885.8	308.2	205.5	99.8
C4	187.7	228.4	227.8	780.8	353.5	671.4	609.1	229.2	469.8	137.1	546.0	583.0	509.9
V1	909.6	837.0	918.2	512.8	930.2	667.7	899.4	453.1	137.3	144.5	243.6	812.8	729.7
V2	269.3	494.7	737.0	893.1	261.9	664.8	668.1	463.7	468.2	575.7	134.6	851.8	757.1
V3	446.2	404.8	168.0	798.1	657.6	214.1	891.9	431.5	727.2	596.2	606.6	532.1	900.4
V4	901.2	813.0	349.0	880.6	921.6	739.2	322.6	263.5	88.9	531.6	165.9	571.3	91.0
M1	539.4	383.2	161.6	599.5	722.7	779.7	696.8	145.8	710.5	54.1	282.6	669.7	854.2
M2	130.7	704.6	915.3	852.7	591.6	874.3	475.3	257.6	854.1	219.6	237.6	191.4	117.1
M3	324.9	711.4	876.4	815.8	872.5	857.5	820.0	770.7	320.9	775.7	573.5	291.3	107.9
M4	923.3	484.9	539.0	797.8	168.1	316.5	279.2	89.9	546.1	715.3	76.1	86.8	773.3

Table S2 Unidirectional estimates of M of Sogatella furcifera populations in the Greater Mekong Subregion (GMS) (Continued)

Population, *i*	L6→*i*	L7→*i*	L8→*i*	T1→*i*	T2→*i*	C1→*i*	C2→*i*	C3→*i*	C4→*i*	V1→*i*	V2→*i*	V3→*i*	V4→*i*
JP	863.6	244.4	616.5	898.6	629.2	100.4	428.1	278.6	631.9	904.4	213.1	240.3	80.6
KY	237.0	832.5	899.7	137.8	556.1	355.6	464.5	170.0	289.5	61.9	890.9	558.0	793.3
YS	188.2	128.8	598.1	801.6	468.1	308.8	377.3	220.5	643.2	489.7	749.1	500.0	172.5
FN	883.2	950.4	681.4	824.7	498.6	199.6	812.9	828.3	440.3	373.4	800.7	827.0	844.2
MD	904.9	712.1	559.2	550.8	833.0	677.4	684.3	327.7	907.0	724.4	676.7	390.9	793.1
BS	741.1	600.3	293.1	420.4	754.6	813.2	315.9	837.1	870.1	780.5	178.2	875.3	892.1
MS	909.8	360.5	645.7	127.5	805.4	302.7	272.4	236.3	490.9	574.8	940.2	493.5	521.5
YIJ	420.2	551.0	785.4	703.6	259.4	103.0	471.1	821.6	253.3	847.3	307.7	654.2	489.6
LC	899.9	855.8	834.6	848.5	334.9	885.8	852.4	550.1	352.1	715.0	229.2	282.3	686.6
NE	646.6	877.7	676.7	358.3	907.3	762.2	186.4	560.2	796.9	863.9	454.8	469.5	833.1
MH	749.8	707.5	82.4	774.9	695.2	801.8	132.0	440.1	185.7	415.4	403.7	237.5	592.1
SJ	692.2	164.9	934.6	843.3	87.1	315.8	268.6	151.9	606.0	211.3	177.6	158.0	91.9
GM	653.5	438.2	680.9	299.4	69.1	801.0	456.0	876.3	469.3	570.8	487.9	311.5	645.6
CY	263.2	543.5	334.9	642.4	165.2	522.9	128.8	532.1	143.2	844.8	562.6	119.3	149.6
CX	887.1	289.6	637.7	596.5	301.9	902.7	826.9	558.8	455.6	739.6	235.9	854.4	920.0
SM	919.0	696.2	820.6	771.8	811.8	225.2	464.5	364.7	777.0	665.7	679.2	546.3	295.7
XP	862.8	874.0	717.0	586.9	889.1	546.1	270.4	502.0	374.4	419.3	715.8	295.5	840.1
YUJ	756.9	322.3	917.0	761.3	925.6	874.0	167.3	599.3	449.3	446.9	557.8	444.3	215.7
SY	132.8	185.0	872.6	874.4	551.4	909.9	545.7	176.2	470.9	408.6	560.1	882.1	686.4
SZ	875.5	444.0	108.1	421.9	252.2	735.7	524.6	100.1	410.0	299.2	825.4	164.7	770.1
L1	277.9	324.2	463.1	866.6	573.4	470.0	363.9	802.4	887.8	177.4	823.5	79.0	271.1
L2	747.6	501.1	857.4	187.9	807.0	870.3	598.8	582.6	306.1	884.4	764.0	87.8	386.6
L3	876.5	746.4	816.1	467.7	705.9	786.1	739.6	314.2	885.7	462.5	318.4	572.7	735.2
L4	909.9	726.7	425.0	135.0	732.9	762.6	294.2	354.7	808.6	498.4	513.0	260.5	836.6
L5	402.0	478.4	671.6	444.0	255.6	134.3	124.2	753.1	676.9	161.0	109.0	579.7	877.8
L6	-	747.0	412.3	159.7	95.1	887.7	135.9	902.4	437.4	201.1	127.2	195.3	469.3
L7	179.7	-	893.2	864.1	724.2	301.9	414.0	894.3	839.9	529.2	430.7	917.4	805.3
L8	778.7	158.8	-	83.6	237.8	295.1	790.5	815.7	288.8	559.1	375.0	61.5	286.7
T1	732.9	243.9	730.7	-	331.4	312.9	496.8	141.5	239.5	114.1	366.7	89.1	379.4
T2	369.9	329.8	451.2	815.0	-	548.7	831.3	867.0	621.6	526.1	781.9	827.5	894.4
C1	787.8	602.4	440.6	272.9	366.3	-	98.2	333.6	681.9	302.6	220.3	374.5	793.6
C2	483.8	153.2	742.6	116.9	164.3	567.1	-	491.7	452.5	658.9	159.4	859.6	406.2
C3	420.0	248.4	739.6	159.4	582.1	143.1	109.3	-	212.8	271.4	255.0	94.8	456.2
C4	241.0	189.5	126.6	273.6	140.9	292.1	124.2	294.1	-	741.5	171.5	723.2	723.6
V1	408.5	880.5	171.4	283.1	262.6	577.1	637.1	609.5	404.9	-	641.6	782.9	544.4
V2	611.8	606.1	459.9	492.9	872.6	773.5	420.0	730.6	423.4	752.5	-	338.5	912.2
V3	355.4	528.6	90.5	558.9	530.5	540.4	766.0	231.7	696.7	882.1	741.6	-	212.7
V4	81.0	161.8	56.4	495.0	186.5	120.7	353.9	66.0	263.8	847.2	252.5	190.2	-
M1	83.7	622.5	144.1	162.7	561.8	131.5	433.0	572.5	865.0	479.3	155.8	313.8	158.8
M2	812.6	832.6	181.9	366.3	97.9	341.5	98.7	143.8	202.1	183.8	50.0	192.2	160.4
M3	556.8	187.7	858.9	415.2	701.5	377.5	720.6	897.7	640.4	854.2	688.2	753.9	537.2
M4	199.3	796.2	854.0	153.2	94.5	147.4	861.8	841.4	332.1	504.6	465.8	612.0	242.6

Table S2 Unidirectional estimates of M of Sogatella furcifera populations in the Greater Mekong Subregion (GMS) (Continued)

Population, *i*	M1→*i*	M2→*i*	M3→*i*	M4→*i*	Total *i*
JP	835.9	436.3	709.9	89.3	7093.1
KY	492.2	389.0	488.7	833.5	3825.3
YS	774.2	627.1	130.0	166.9	7081.4
FN	866.7	622.9	665.8	138.7	6056.0
MD	602.0	740.9	185.5	591.9	5422.4
BS	810.1	908.6	551.1	188.0	6633.9
MS	826.1	811.9	885.1	920.5	7126.7
YIJ	368.4	902.8	458.1	656.9	6604.0
LC	204.5	408.9	357.5	714.0	6891.8
NE	929.0	567.1	341.1	852.0	4826.2
MH	106.4	249.0	370.1	652.8	6617.6
SJ	68.0	173.5	333.3	197.0	5469.9
GM	429.8	284.9	741.3	389.4	5137.9
CY	251.0	154.2	277.7	220.4	6306.6
CX	279.4	915.8	806.9	789.9	8604.8
SM	884.4	789.6	535.0	654.7	4879.6
XP	919.1	689.0	500.0	459.0	7592.4
YUJ	708.2	766.3	233.2	665.5	7773.5
SY	729.6	389.6	195.7	338.5	7499.6
SZ	332.0	82.2	327.6	641.4	5355.7
L1	424.4	533.6	814.0	413.0	6112.2
L2	671.1	684.4	858.4	891.4	7882.8
L3	907.0	162.1	732.9	855.5	5424.4
L4	564.3	142.1	394.9	627.2	7721.8
L5	93.2	174.6	190.1	826.2	6760.8
L6	643.1	126.4	668.4	325.1	3930.5
L7	867.8	890.2	904.2	120.3	4252.6
L8	410.0	222.7	390.4	492.9	7467.5
T1	233.8	48.6	236.1	858.9	4966.0
T2	885.0	774.5	830.5	264.9	4882.7
C1	657.2	296.4	153.2	628.9	6973.7
C2	142.9	391.8	384.2	808.2	4623.6
C3	681.3	331.5	426.7	252.3	4871.6
C4	726.0	823.9	124.7	711.5	4110.8
V1	547.7	68.4	584.7	142.2	7307.1
V2	681.6	775.9	263.7	643.0	8130.7
V3	640.1	273.3	51.4	295.9	8087.2
V4	442.5	187.2	754.6	173.1	4736.9
M1	-	426.7	339.9	512.2	4522.1
M2	500.4	-	485.6	763.4	3831.2
M3	636.6	838.9	-	267.3	7836.2
M4	368.1	849.3	97.0	-	4637.9
